# Kidney disease characteristics, prevalence, and risk factors in León, Nicaragua: a population-based study

**DOI:** 10.1186/s12882-023-03381-1

**Published:** 2023-11-12

**Authors:** Anna Strasma, Ángel Mejía Reyes, Aurora Aragón, Indiana López, Lawrence P. Park, Susan L. Hogan, Nathan Thielman, Christina Wyatt, Marvin González-Quiroz

**Affiliations:** 1 Department of Medicine, Division of Nephrology, Duke University School of Medicine, Durham, NC USA; 2 Duke Global Health Institute, Durham, NC USA; 3 Bioanalysis and Immunology Research Group, National Autonomous University of Honduras, Tegucigalpa, Honduras; 4 WUQU’ KAWOQ, Maya Health Alliance, Chimaltenango, Guatemala; 5 Department of Medicine, Division of Infectious Diseases, Duke University School of Medicine, Durham, NC USA; 6 Department of Medicine, Division of Nephrology and Hypertension and the UNC Kidney Center, University of North Carolina – Chapel Hill, Chapel Hill, NC USA; 7 Duke Clinical Research Institute, Durham, NC USA; 8 Department of Renal Medicine, University College London, London, UK; 9 School of Medicine, Universidad Nacional de Chimborazo, Riobamba, Ecuador

**Keywords:** CKDu, Mesoamerican nephropathy, Risk factors, Nicaragua

## Abstract

**Background:**

CKD of unknown etiology (CKDu) disproportionately affects young people in Central America who lack traditional CKD risk factors (diabetes and hypertension) and has instead been variably linked to heat stress, occupational and environmental exposures, nephrotoxic medications, and/or genetic susceptibility. This study aimed to estimate the prevalence of CKD and identify risk factors for traditional CKD and CKDu in Nicaragua.

**Methods:**

Surveys and assessment for CKD markers in urine and serum were performed in 15–59 year olds in households of the León municipality of Nicaragua. The survey included questions on demographics, health behaviors, occupation, and medical history. Participants with CKD were subdivided into traditional CKD and suspected CKDu based on history of diabetes, hypertension, or other specified conditions. A multinomial logistic regression model was used to identify factors associated with traditional CKD and suspected CKDu, compared to the non-CKD reference group.

**Results:**

In 1795 study participants, CKD prevalence was 8.6%. Prevalence in males was twofold higher than females (12% vs 6%). Of those with CKD, 35% had suspected CKDu. Both traditional CKD and CKDu were associated with male sex and increasing age. Traditional CKD was associated with a family history of CKD, history of urinary tract infections, and lower socioeconomic status, while CKDu was associated with drinking well water and a lower body mass index.

**Conclusions:**

Both traditional CKD and CKDu are significant burdens in this region. Our study supports previous hypotheses of CKDu etiology and emphasizes the importance of CKD screening.

**Supplementary Information:**

The online version contains supplementary material available at 10.1186/s12882-023-03381-1.

## Background

Chronic kidney disease (CKD) is expectedto be the fifth leading cause of mortality worldwide by 2040 [1]. CKD prevention requires increased awareness and identification of the underlying causes, which may vary globally. In some regions, in particular Central America and Southeast Asia, unexpectedly high rates of CKD affect young populations without traditional risk factors, a condition commonly known as CKD of unknown etiology (CKDu) [2]. Epidemiological studies have described CKDu as a form of chronic interstitial nephritis without hematuria or proteinuria, most commonly affecting males from lowlands working in agriculture [3, 4].

The etiology of CKDu is unknown but is likely to be multifactorial. Leading theories include heat stress, recurrent volume depletion, and hyperuricemia worsened by consumption of fructose [5–7]. Uric acid is released from muscle during strenuous labor and can crystallize in the urine causing tubular damage [8]. Other hypothesized contributors include agrochemical or heavy metal exposures, which could occur during work or from the environment, such as well water contamination [4, 9–12]. Nephrotoxic medications such as non-steroidal anti-inflammatory drugs (NSAIDs) and diuretics are commonly used for many symptoms, including dysuria and lower abdominal pain colloquially thought to be due to urinary tract infection [13, 14]. Tropical infectious diseases such as leptospirosis, hantavirus, or malaria have also been proposed as potential contributors to CKDu [15–17]. Finally, a genetic predisposition to CKDu has been hypothesized due to familial clustering [18].

The prevalence of CKDu is challenging to estimate due to a lack of large community-based studies in Central America. In Nicaragua, the mortality from CKD is high and increasing, at least partly attributed to CKDu, highlighting the need for further studies to define its prevalence and risk factors [19]. This study aimed to estimate the prevalence of CKD and to identify possible risk factors for traditional and non-traditional CKD in an area of Nicaragua where the presence of CKDu has been established [4].

## Methods

### Setting and participants

Our study took place in the municipality of León, with a population of 204,000 at the time of this study [20]. Households were randomly selected from the León Health and Demographic Surveillance System (LHDSS), which is designed to be representative of the entire municipality, and household members aged 15 to 59 years old were recruited between May and August 2014 [21]. Household visits occurred on weekends to limit absences due to work. Data were collected by staff of the Research Center of Health, Work and Environment (CISTA), the Center for Demography and Health Research (CIDs) and a previously trained group of medical students from Universidad Nacional Autonoma de Nicaragua, León (UNAN-León). The current analysis excluded participants who did not have a serum creatinine sample collected.

### Procedures

Data were collected through a standardized questionnaire, physical measurements, and collection of blood and urine samples. Weight was measured with a digital scale, calibrated daily, and height with a measuring rod. These values were used to calculate body mass index (BMI).

Serum creatinine, blood urea nitrogen (BUN), and uric acid were measured in the national laboratory of the Nicaraguan Ministry of Health (MINSA) using the Roche Cobas Integra 400 Plus. The instrument was calibrated for creatinine analysis using isotope dilution mass spectrometry reference standards. Urine samples were analyzed through dipstick testing (Combur test). Participants with abnormal results were routed to local medical services provided by MINSA for further care.

### Variable definitions

Socioeconomic level was based on the World Bank’s Development Research group, using the daily per capita income, calculated in United States dollars ($) in 2005 [22]. Categories include extremely poor (< $1.25), poor ($1.25—$2.00), and not poor (≥ $2.01) [22].

Current and previous occupations were classified using the United Nations' International Standard Industrial Classification of All Economic Activities [23]. The cumulative years of working in any agricultural occupation were calculated. For the subgroup of females who worked in agriculture, work tasks were reviewed in detail to evaluate for the performance of hard manual labor.

CKD awareness was considered positive if the participant indicated a personal history of CKD. NSAID or diuretic use was defined as self-reported use for more than one consecutive week.

Estimated glomerular filtration rate (eGFR) was calculated using the CKD Epidemiology Collaboration 2021 Equation [24]. Stages of CKD were defined based on Kidney Disease Improving Global Outcomes (KDIGO) guidelines [25].

### Case definitions

**Fig. 1:**
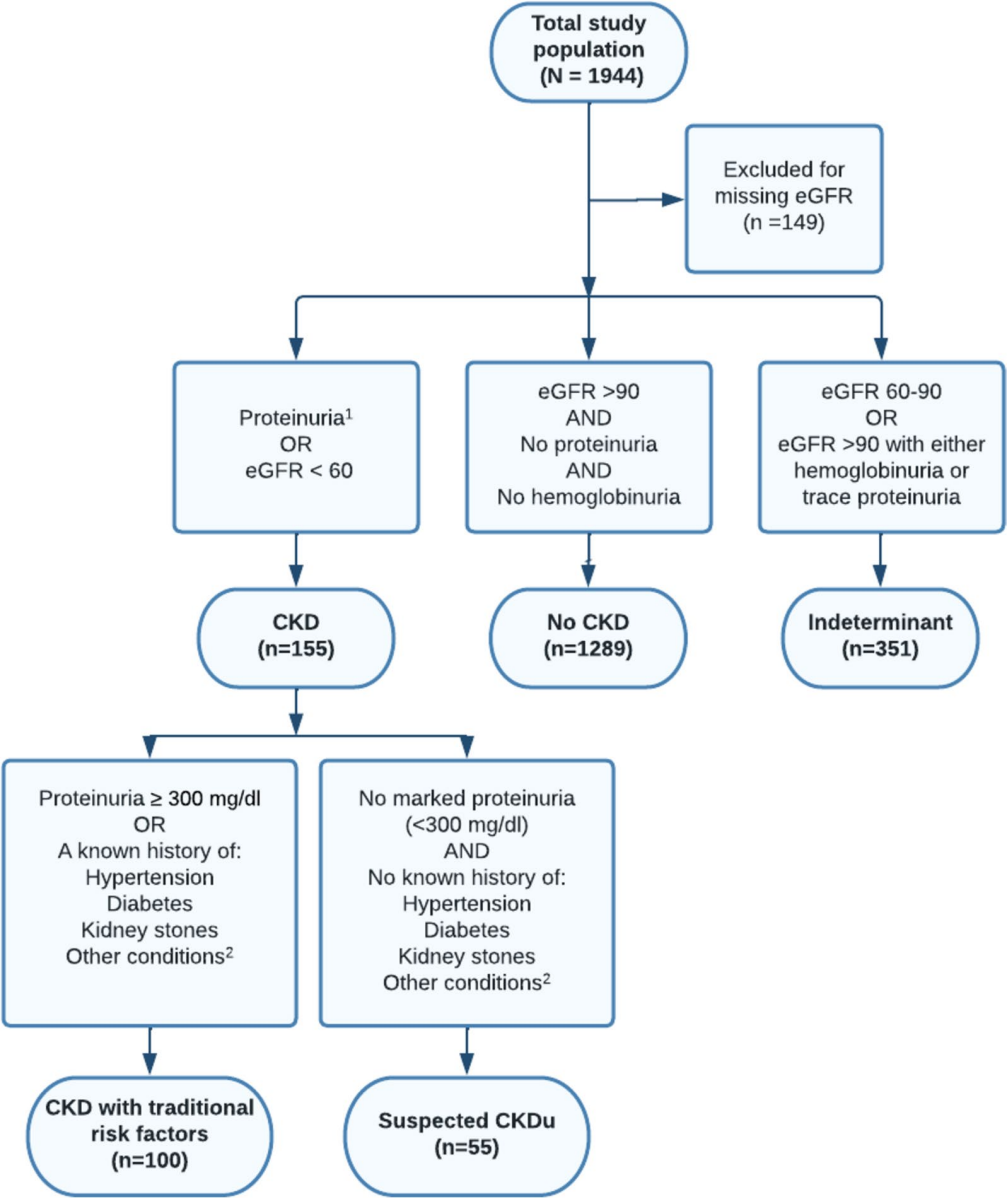
Study participant classification and sample sizes. Footnotes: eGFR units are ml/min/1.73m^2^. 1. Proteinuria is defined by urine dipstick. 2. Other conditions include taking blood pressure medication, glucosuria, hyperglycemia, known history of urologic, hematologic disease, genetic or hereditary kidney disease, or autoimmune disease

The Pan American Health Organization’s (PAHO) clinical case definition for CKDu includes individuals aged 2–59, diagnosed with CKD by KDIGO guidelines and without exclusion criteria including diabetes or hypertension, urologic pathology, primary glomerulopathy, hematologic or autoimmune disease, repeated exposure to contrast media or phosphate-containing products, or hematuria or significant proteinuria indicating a different underlying cause [26]. Participants were classified as suspected CKDu if they matched the PAHO criteria as closely as possible based on the available data, with self-reported diabetes or hypertension considered sufficient to exclude participants from the CKDu classification. The study case definitions included: (a) no CKD, (b) CKD with traditional risk factors, (c) suspected CKDu, and (d) indeterminant CKD. The study case definitions are further illustrated in Fig. 1.

- No CKD: Study participants with an eGFR ≥ 90 ml/min/1.73m^2^ and dipstick negative proteinuria and hemoglobinuria.
- CKD with traditional risk factors (traditional CKD)*:* Study participants with 1) proteinuria ≥ 300 mg/dl on urine dipstick or 2) participants with an eGFR ≤ 60 ml/min/1.73m^2^ who have one or more of the traditional risk factors for CKD including hypertension (or who were taking blood pressure medications), diabetes, hyperglycemia, or kidney stones. Participants with eGFR ≤ 60 ml/min/1.73m^2^ with hemoglobinuria or glucosuria were also included in this group.
- Suspected CKDu: Study participants with eGFR ≤ 60 ml/min/1.73m^2^without the following features: (1) proteinuria ≥ 300 mg/dl or hemoglobinuria on urine dipstick, (2) reported traditional risk factors for CKD [26, 27].
- Indeterminant CKD: For the purpose of comparison between groups that clearly have impaired kidney function and those that do not, comparative analyses excluded participants with (1) eGFR between 60 and 90 ml/min/1.73m^2^ without proteinuria (2) eGFR ≥ 90 ml/min/1.73m^2^ with hemoglobinuria or trace proteinuria.

### Analysis

Continuous variables were described in medians and quartiles while categorical variables were described in proportions. Proportions were calculated excluding participants with missing data for that particular covariate. Participants categorized as indeterminant for CKD were included in the denominator for CKD prevalence estimates but were excluded from analyses comparing traditional CKD and suspected CKDu. Covariates were compared between the following groups: no CKD, traditional CKD, and suspected CKDu. Nonparametric tests were used due to small sample size in the CKD groups. Continuous variables were compared with Kruskal Wallis test and categorical variables were compared with Fisher’s Exact test. Significance values were set at α = 0.05.

Multinomial logistic regression was used to evaluate the odds ratio (OR) and 95% confidence intervals (CI) for associations with CKD. Sex, age, and socioeconomic status have been shown to be associated with CKD and were selected as covariates for the model. Variables found to be significant in the univariate analysis that had at least a 15% prevalence were added individually to the model with the aforementioned prioritized variables and evaluated. Variables with a *p*-value < 0.05 were included in a full model which was reduced by backward elimination. If covariates were strongly correlated, then the covariate with the smaller *p*-value was chosen. The ORs for age and BMI were calculated in the model as a per-year or per-unit increase respectively, but then exponentiated to report the OR for a 10-year increase in age and a 5-unit increase in BMI. A variance inflation factor was calculated in the full model to ensure there was no influence of multicollinearity. All analyses were conducted using R language for statistical computing (version 4.2.1) [28].

In separate sensitivity analyses, we evaluated the impact of (1) including the indeterminant CKD group as an additional group in comparative analyses or (2) including the indeterminant CKD group as part of the no CKD group.

### Human subjects research protections

This study was approved by the ethics committee of the medical sciences at UNAN-León, and analysis of deidentified data was determined exempt from full review by the Duke University Institutional Review Board. Written informed consent was obtained from each adult participant, and both parental consent and participant assent were obtained for those < 18 years old.

## Results

After excluding 149 participants with missing eGFR, the study population included 1795 participants (Fig. 1). Females made up 60% of the population, and the median age was 36 (Table 1).

**Table 1:** Total study population demographics

Demographics	Total study population (*N* = 1795)
Sex	
Male	714 (40%)
Female	1081 (60%)
Median age, years (IQR)	36 (25–47)
Age categories	
< 20 years old	210 (12%)
20–39 years old	872 (49%)
40–59 years old	713 (40%)
Socioeconomic level^a^	
Extremely poor	595 (33%)
Poor	462 (26%)
Not poor	721 (41%)
Zone	
Rural	619 (34%)
Urban	1176 (66%)
Illiterate	136 (7%)
Last grade level completed, median (IQR)^b^	9 (6–12)

*Abbreviations*: *IQR* Interquartile range

^a^Socioeconomic level based on the World Bank’s Development Research group. Missing socioeconomic status on 17 participants

^b^Missing grade level for 124 participants

### CKD prevalence

The overall prevalence of CKD was 8.6% (Table 2). Males had a higher prevalence of CKD than females (12.5% vs. 6.1%). CKD was based on proteinuria alone in 30% of cases. Stage 3 CKD was most common (46% of CKD), while Stages 4 and 5 CKD were less common (16% and 8% respectively) with a much higher proportion of males in these higher stages. Of the 155 participants with CKD, 100 (65%, or 5.6% overall prevalence) had high-grade proteinuria or traditional CKD risk factors while 55 (35%, or 3.1% overall prevalence) were classified as suspected CKDu.

**Table 2:** Prevalence of CKD and CKD awareness

	Overall, n (%) *N* = 1795	Males, n (%) *n* = 714	Females, n (%) *n* = 1081
**Population with CKD**	155 (8.6%)	89 (12.5%)	66 (6.1%)
Stage of CKD^a^			
Proteinuria with eGFR > 60^b^	47 (30.3%)	18 (20.2%)	29 (43.9%)
Stage 3	71 (45.8%)	39 (43.8%)	32 (48.5%)
Stage 4	25 (16.1%)	21 (23.6%)	4 (6.1%)
Stage 5	12 (7.7%)	11 (12.4%)	1 (1.5%)
Self-reported history of CKD (CKD Awareness)	34 (21.9%)	22 (24.7%)	12 (18.2%)
CKD awareness in each stage of CKD			
Proteinuria with eGFR > 60^b^	3 (6.4%)	1 (5.6%)	2 (6.9%)
Stage 3	16 (22.5%)	7 (17.9%)	9 (28.1%)
Stage 4	7 (28%)	6 (28.6%)	1 (25.0%)
Stage 5	8 (66.7%)	8 (72.7%)	0
CKD category			
CKD with traditional risk factors	100 (64.5%)	52 (58.4%)	48 (72.7%)
Suspected CKDu	55 (35.5%)	37 (41.6%)	18 (27.3%)

*Abbreviations*: *CKD* Chronic kidney disease, *eGFR* Estimated glomerular filtration rate, *CKDu* Chronic kidney disease of unknown etiology

^a^CKD stages by Kidney Disease Improving Global Outcomes (KDIGO)

^b^eGFR in ml/min/1.73 m^2^

Only 22% of participants with CKD reported a personal history of CKD (Table 2). Although awareness increased at higher stages of CKD, 4 participants with Stage 5 CKD and 18 with Stage 4 CKD did not report a history of CKD. Awareness was low among participants with both traditional CKD (25%) and suspected CKDu (16%).

### Comparison of groups with no CKD, CKD from traditional causes, and suspected CKDu

The groups differed significantly in most demographic variables. We observed a higher proportion of males in both the traditional CKD group (52%) and the suspected CKDu group (67%) compared to the non-CKD group (40%) (Table 3). The CKD groups were older (median age 48 years for traditional CKD and 44 years in CKDu) than the non-CKD group (33 years) and higher proportions classified as extremely poor, or poor compared to the non-CKD group. Illiteracy rate was 20% in the CKDu group versus 13% in the traditional CKD group and 6% in the non-CKD group. A higher proportion of the suspected CKDu group lived in rural zones, and 38% obtained water from a well compared to 29% in the traditional CKD group and 19% in the non-CKD group. Reported daily water intake was highest in the suspected CKDu group, but soda intake, current alcohol use, and history of drug use did not differ between groups. NSAID and diuretic use was highest in the traditional CKD group. Exercise and eating fresh foods were rare overall.

**Table 3:** Comparison of demographics, health behaviors, medical history, and occupation by CKD category (no CKD, CKD with traditional risk factors, and suspected CKDu)

Characteristics	No CKD (*n* = 1289)	CKD with traditional risk factors (*n* = 100)	Suspected CKDu group (*n* = 55)	*P* value
**Demographics**				
Sex				< 0.001^a^ *
Male	513 (39.8%)	48 (48.0%)	37 (67.3%)	
Female	776 (60.2%)	52 (52.0%)	18 (32.7%)	
Age, median (IQR)	33 (23–43)	48 (37–54)	44 (34–55)	< 0.001^b^ *
Socioeconomic level				0.029^a^ *
Extremely poor	421 (33.0%)	43 (43.0%)	24 (43.6%)	
Poor	328 (25.7%)	29 (29.0%)	13 (23.6%)	
Not poor	527 (41.3%)	28 (28.0%)	16 (29.1%)	
Illiterate	75 (5.8%)	13 (13.0%)	11 (20.0%)	< 0.001^a^ *
Highest grade level completed, median (IQR)^c^	9 (6–12)	6 (3–11)	6 (4–9)	< 0.001^a^ *
Rural Zone	447 (34.7%)	37 (37.0%)	24 (43.6%)	0.352^a^
Well water source^d^	242 (18.8%)	29 (29.0%)	21 (38.2%)	< 0.001^a^ *
**Health behaviors**				
Daily water intake (L), median (IQR)	2 (1–3)	2 (1–4)	3 (2–4)	0.003^b^ *
Daily soda intake (L), median (IQR)	0.25 (0–0.5)	0 (0 – 0.5)	0 (0–0.5)	0.129^b^
Alcohol consumption in last day	80 (6.2%)	2 (2.0%)	7 (12.7%)	0.078^b^
History of smoking	285 (22.1%)	27 (27.0%)	19 (34.5%)	0.061^a^
Median years smoking	8 (3–19)	22 (13–35)	24 (16–35)	< 0.001^b^ *
History of drug consumption	121 (9.4%)	12 (12.0%)	6 (10.9%)	0.562^a^
Exercise ≥ 2 times weekly	256 (19.9%)	14 (14.0%)	7 (12.7%)	0.192^a^
Eat fruits, vegetables, or salads ≥ 2 times weekly	769 (59.9%)	60 (60.0%)	23 (41.8%)	0.030^a^ *
**Medical history**				
NSAID use	346 (26.8%)	37 (37.0%)	16 (29.1%)	0.083^a^
Diuretic use	49 (3.8%)	13 (13.0%)	2 (3.6%)	< 0.001^a^ *
Hypertension	142 (11.0%)	53 (53.0%)	0	< 0.001^a^ *
Diabetes	52 (4.0%)	22 (22.0%)	0	< 0.001^a^ *
Kidney stones	67 (5.2%)	18 (18.0%)	0	< 0.001^a^ *
Arthritis	106 (8.2%)	27 (27.0%)	8 (14.5%)	< 0.001^a^ *
Malaria	128 (9.9%)	15 (15.0%)	7 (12.7%)	0.196^a^
Urinary tract infection	631 (49.0%)	65 (65.0%)	28 (50.1%)	0.008^a^ *
Family history of CKD	312 (24.2%)	42 (42.0%)	16 (29.1%)	0.003^a^ *
**Occupation**				
Current agricultural occupation^e^	86 (6.7%)	11 (11.0%)	16 (29.1%)	< 0.001^a^ *
History of working in agriculture	228 (17.7%)	27 (27.0%)	25 (45.5%)	< 0.001^a^ *
Males	152 (66.7%)	24 (88.9%)	22 (88.0%)	< 0.001^a^ *
Females	76 (33.3%)	3 (11.1%)	3 (12.5%)	0.411^a^
Years worked, median(IQR)	10 (5–20)	13 (9–30)	20 (8–32)	0.042^b^ *
History of fainting at work	136 (10.6%)	17 (17.0%)	6 (10.9%)	0.139^a^
History of heat exhaustion	445 (34.5%)	42 (42.0%)	15 (27.2%)	0.163^a^

*Abbreviations*: *CKD* Chronic kidney disease, *CKDu* Chronic kidney disease of unknown etiology, *IQR* Interquartile range, *L* Liters, *NSAID* Non-steroidal anti-inflammatory drug

All percentages calculated from non-missing data. Less than 5% of each group is missing data unless otherwise indicated

^a^*p*-value calculated with Fisher’s Exact Test

^b^*p*-value calculated with Kruskal Wallis test

^c^92 participants missing grade level

^d^The remainder of participants obtained water from a public water system with interior plumbing with the exception of 4 participants in the No CKD group who had a different water source that did not fit either of these categories (i.e. river, purchasing drinking water, ect)

^e^Of those with a current occupation in agriculture, all were male except 4 females in the no CKD group

* significant finding at *p*-value < 0.05

Drinking well water was reported in 296 participants in the included groups. Almost half (47%) of participants using well water reported no treatment, and 48% reported using only chlorination treatment for their well water. Only 14 (5%) reported boiling, using a household filter, or other treatment to their drinking water. Type of water treatment did not differ significantly between the group with traditional CKD, CKDu, and no CKD (*p* = 0.85). Most of the wells (175, 75% of wells with data available) were hand-dug, while 25% were machine-drilled, and this did not differ by CKD category. There were 113 (39%) wells reported to be close to agricultural fields, which did not differ between groups (*p* = 0.92). The most common reported crops were peanuts (44), corn (21), sugar cane (19), sesame (15), and yuca (16). This did not differ between CKD categories except sugar cane, which was proportionally more common in the traditional CKD group than the others (*p* = 0.045).

The distribution of diabetes, hypertension, and kidney stones history largely reflects the study design (Table 3). Self-reported history of urinary tract infections (UTIs) was more common in the traditional CKD (65%) group than the suspected CKDu (50%) and the non-CKD groups (49%). Participants with a history of UTIs had a median of one infection in the past year, and a notable 41% of males reported a history of UTIs. Family history of CKD was highest in the traditional CKD group and almost equivalent in the suspected CKDu and non-CKD group.

The most common current occupation was homemaker for females (42% of females) and agricultural worker or student for males (18% and 17% of males, respectively). The suspected CKDu group had the highest proportions currently working in agriculture and with a lifetime history of working in agriculture compared to the other groups (Table 3). The suspected CKDu group also had the longest median time of working in agriculture (20 years). History of fainting or experiencing heat exhaustion at work did not differ between groups. Among 112 females with a history of working in agriculture, there were 3 with traditional CKD and 3 with suspected CKDu. Many females (66) performed tasks that included cutting crops or wood, with the largest number cutting cotton. Other tasks females performed commonly were seeding and weeding. Self-reported history of heat exhaustion was similar between male and female agricultural workers.

Median BMI was highest in the traditional CKD group (28) and lowest in the suspected CKDu group (24) (Table 4). Overall, there was a high prevalence of overweight, obese, or extremely obese individuals. The CKDu group had the highest uric acid levels in both males and females. When results were stratified by CKD stage, uric acid was still higher in the CKDu group, although the difference was no longer significant (Supplemental Figs. 1 and 2).

**Table 4:** Health assessment and measurements by CKD category

Health assessment	No CKD (*n* = 1289)	CKD with traditional risk factors (*n* = 100)	Suspected CKDu group (*n* = 55)	*P* value
BMI categories				0.046^a^ *
Underweight (< 18.5)	46 (3.6%)	3 (3.0%)	1 (1.8%)	
Normal (18.5 – 24.5)	427 (33.4%)	28 (28.0%)	28 (50.1%)	
Overweight (25–30)	442 (34.6%)	38 (38.0%)	14 (25.4%)	
Obese (30–34.5) or extremely obese (> 35)	363 (28.4%)	33 (33.0%)	10 (18.1%)	
BMI, median (IQR)	27 (23–31)	28 (24–31)	24 (22–28)	0.004^b^ *
Serum uric acid (umol/L), median (IQR)				
Males (reference range 220–476)^c^	321 (274 – 371)	435 (360—508)	523 (430 – 589)	< 0.001^b^ *
Females (reference range 161–363)^c^	243 (206 – 249)	335 (245 – 400)	359 (288 – 500)	< 0.001^b^ *
Serum BUN (mmol/L), median (IQR)	3.0 (2.4 – 3.7)	5.5 (3.9 – 9.0)	7.1 (5.5 – 9.4)	< 0.001^b^ *
Serum Creatinine (umol/L), median (IQR)	59 (51—69)	127 (69 – 184)	172 (127 -236)	< 0.001^b^ *
eGFR (ml/min/1.73 m^2^), median (IQR)	120 (110–129)	51 (36–105)	40 (25–54)	< 0.001^b^ *
CKD Stages				< 0.001^a^ *
Proteinuria with eGFR > 60 ml/min/1.73 m^2^	0	39 (39.0%)	8 (14.5%)	
Stage 3	0	42 (42.0%)	29 (52.7%)	
Stage 4	0	12 (12.0%)	13 (23.6%)	
Stage 5	0	7 (7.0%)	5 (9.1%)	
Urine dipstick results				
Positive leukocyte esterase^d^	144 (14.1%)	20 (20.8%)	12 (24.5%)	0.035^a^ *
Positive nitrites^e^	44 (4.3%)	7 (7.3%)	4 (8.1%)	0.144^a^
Proteinuria^f^				< 0.001^a^ *
None	993 (100%)	39 (41.1%)	31 (63.2%)	
Trace	0	8 (8.4%)	9 (18.4%)	
30 mg/dl	0	5 (5.3%)	7 (14.3%)	
100 mg/dl	0	2 (2.1%)	2 (4.1%)	
300 mg/dl	0	36 (37.9%)	0	
1000 mg/dl	0	5 (5.3%)	0	
Current symptoms				
High thirst	676 (52.4%)	57 (57.0%)	28 (50.1%)	0.653^a^
Dysuria	351 (27.2%)	28 (28.0%)	9 (16.4%)	0.203^a^
Dark urine	356 (27.6%)	36 (36.0%)	8 (14.5%)	0.015^a^ *
Cramps	241 (18.7%)	37 (37.0%)	16 (29.1%)	< 0.001^a^ *

All percentages calculated from non-missing data. Less than 5% of each group is missing data unless otherwise indicated

*Abbreviations*: *CKD* Chronic kidney disease, *CKDu* Chronic kidney disease of unknown etiology, *BMI* Body mass index, *IQR* Interquartile range, *BUN* Blood urea nitrogen

^a^*p*-value calculated with Fisher’s Exact Test

^b^*p*-value calculated with Kruskal Wallis test

^c^Reference range from Mayo Clinic Reference Laboratories and units converted [29].

^d^298 participants missing leukocyte esterase data

^e^281 participants missing nitrites data

^f^307 participants missing proteinuria data

^*^significant finding at *p*-value < 0.05

**Fig. 2:**
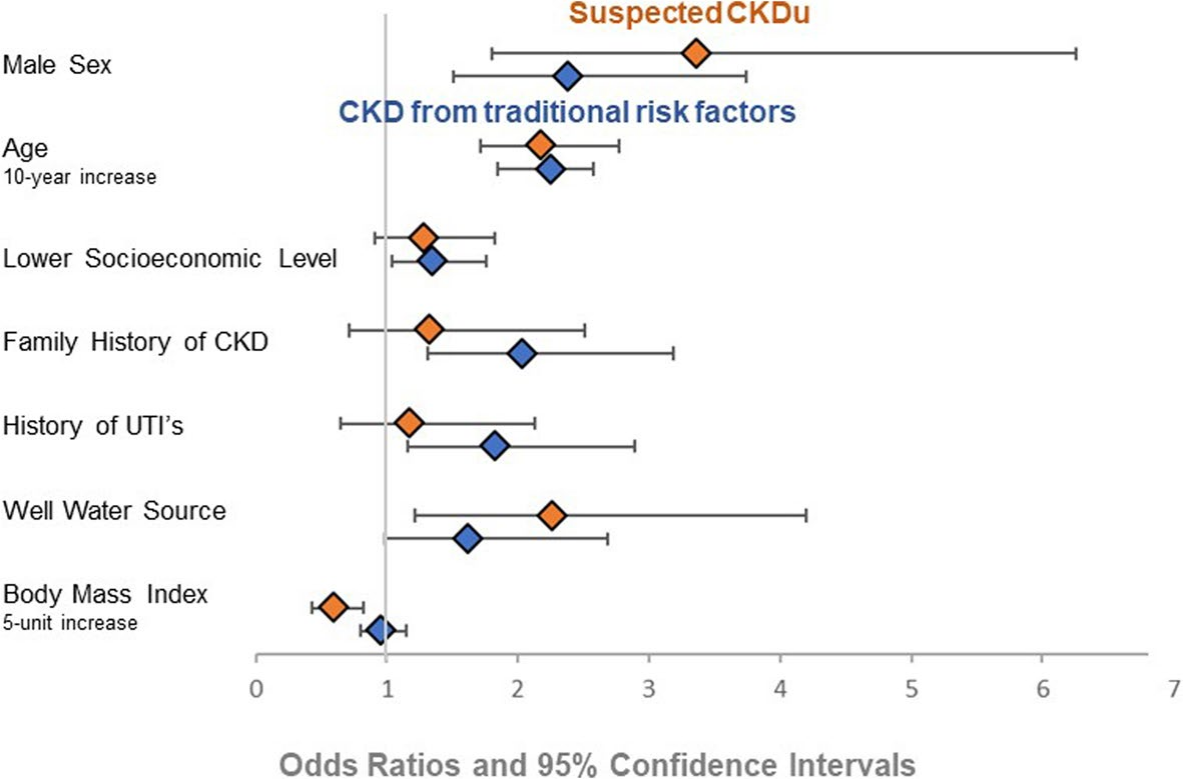
Odds ratios and 95% confidence intervals for the multinomial logistic regression model for CKD with traditional risk factors and suspected CKDu. The reference group is participants with no CKD. Lower socioeconomic status represents a change in one classification per previously defined criteria. Well water source is compared to a public water system. Abbreviations: CKD: chronic kidney disease. CKDu: Chronic kidney disease of unknown etiology. UTI: urinary tract infection. BMI: body mass index

A higher proportion of participants in the suspected CKDu group had CKD Stages 4 and 5, with a median eGFR of 40 ml/min/1.73 m^2^ compared to 51 ml/min/1.73 m^2^ in the traditional CKD group (Table 4). Among 1137 with available data from dipstick urinalysis, almost half of participants in the traditional CKD group have proteinuria (48%) compared to only 16% in the CKDu group. Urinary leukocyte esterase was common (> 20%) in both CKD groups.

The final multinomial logistic regression model included the prioritized covariates (sex, age, socioeconomic status), plus BMI (as a continuous variable), water source (well or public water system), family history of CKD (yes or no), and history of UTI (yes or no) (Fig. 2). Variance inflation factors were all < 1.2, indicating no collinearity of covariates in the final model. Male sex was associated with increased odds of both traditional CKD (OR 2.38, CI 1.51–3.74) and suspected CKDu (OR 3.36, CI 1.80–6.26). A 10-year increase in age was associated with an increased odds of both traditional CKD (OR 2.25, CI 1.85–2.75) and suspected CKDu (OR 2.18, CI 1.71–2.77). Lower socioeconomic level was associated with higher odds of traditional CKD (OR 1.35, CI 1.04–1.76), but was not significantly associated with suspected CKDu (OR 1.29, CI 0.91–1.82). A family history of CKD was strongly associated with higher odds of traditional CKD (OR 2.04, CI 1.31–3.19) but was not significantly associated with suspected CKDu (OR 1.33, CI 0.71–2.51). Similarly, a history of UTIs were associated with traditional CKD (OR 1.83, CI 1.16–2.89) but not with suspected CKDu (OR 1.18, CI 0.65–2.13). Having a well as opposed to a public water system increased the odds of suspected CKDu (OR 2.26, CI 1.21–4.20) but was not significantly associated with traditional CKD (OR 1.62, CI 0.98–2.68). A five unit increase in BMI was associated with 0.4 lower odds of developing CKDu (OR 0.60; CI 0.43–0.82), while BMI was not significantly associated with traditional CKD (OR 0.96; CI 0.80–1.15).

The indeterminant CKD group (*n* = 351) was excluded from the above analysis, but we performed an analysis for both 1) the indeterminant group included as an outcome and 2) the indeterminant group included in the no CKD group (Tables S1, S2, S3 and S4). We developed a multinomial logistic regression model for both analyses, and found the odds ratios were similar for most variables, but the covariate of agricultural work history remained significant and was included in the final model when the indeterminant group was included as an outcome (Tables S5 and S6). History of agricultural work was protective for traditional CKD (OR 0.52 CI 0.29–0.92) when indeterminant CKD group is included as a separate group (Table S5).

## Discussion

This study identified a CKD prevalence of 8.6% in the León municipality of Nicaragua, with a twofold higher prevalence among males versus females. This trend is consistent with most prior studies of CKDu in Central America, however, it is notable that many of those studies were focused on agricultural workers [4, 30]. One-third of participants with CKD lacked traditional risk factors, leading to an overall prevalence of suspected CKDu of 3.1% in this young population. The odds of suspected CKDu were 3 times higher for males and 2 times higher for participants with a well drinking water source. Older age and lower BMI were also associated with CKDu.

Our observed prevalence of CKD is similar to a previous report by Lebov et al., who demonstrated a CKD prevalence of 7.5% with marked male predominance four years earlier in the same municipality [31]. Unlike the previous study, our analysis was restricted to participants age 15–59 years old and included albuminuria in the diagnosis of CKD. The observed prevalence of CKD is much higher than expected given the younger age distribution. For example, the observed prevalence of CKD in participants age 20–39 is 10 times higher, and the prevalence in those age 40–59 years is 3 times higher, compared to these populations in the United States National Health and Nutritional Examination Survey (2011–2014) [32].

A low proportion of participants with CKD reported a personal history of CKD. Although it is possible that some cases that we classified as Stage 1–3 CKD would not be confirmed on repeat testing, awareness of CKD was also low among participants with more advanced CKD. This may be due to poor healthcare provider communication or due to inadequate screening. Additional efforts should be taken to support healthcare providers in counseling patients on the diagnosis of CKD, preventive measures against disease progression, and estimated individual risk of kidney failure. Population-level and workplace health education could be considered, particularly in high-risk areas such as Nicaragua, because awareness is an essential component of advocacy for workers’ rights and implementation of better worker conditions [33]. CKD screening should not be limited to only those with traditional risk factors, as 35% of the participants with CKD would not have been screened using this limited approach. Screenings of the entire population in certain areas based on high prevalence in prior studies or suspected risk factors should be considered.

**Fig. 3:**
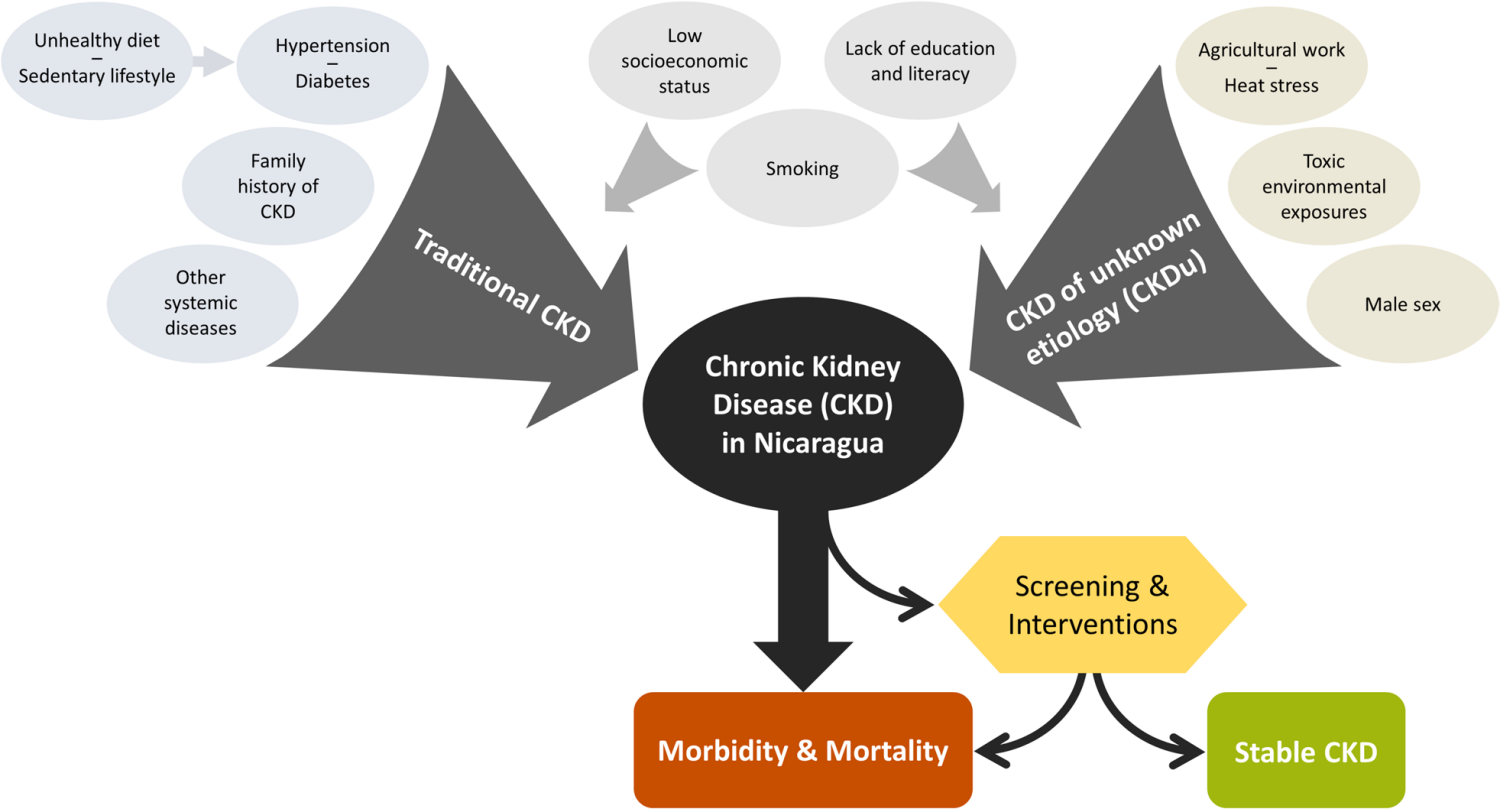
Conceptual map of the contributors to CKD in Nicaragua. Abbreviations: CKD: Chronic kidney disease. CKDu: Chronic kidney disease of unknown etiology

Among participants with CKD in our study, 65% had traditional risk factors and 35% had suspected CKDu. This suggests that this region has two different pathways leading to a public health crisis: both the rise in traditional risk factors, such as diabetes and hypertension, and CKDu (Fig. 3). For each pathway, it is key to determine risk factors, implement preventative programs, and provide early intervention to prevent the devastating mortality and morbidity caused by CKD in Nicaragua.

We considered our study findings in light of proposed hypotheses for the etiologies of CKDu. Obtaining drinking water from a well was associated with a remarkable 2.3 times higher odds of suspected CKDu. Many wells (39%) were located near agricultural fields, and participants using wells most commonly did not perform any water treatment or only used chlorine, which would not protect against chemical environmental toxins. In addition, CKDu was associated with a history of agricultural occupation. These findings suggest toxicity from agrochemicals or heavy metals could play a role in CKDu development. Large population-based studies have identified an association of CKDu with agrochemical exposures, and a specific tubulotoxic mechanism has been proposed based on CKDu biopsy pathology [34–36]. Future research should focus on investigation of environmental contaminants.

CKDu was associated with agricultural occupation in univariate analysis, but after adjusting for sex, age, and socioeconomic status, the association of CKDu with agricultural work was no longer significant. Examination of agricultural occupation tasks showed over half of women had a history of heavy manual labor, such as cutting cotton, and a similar proportion of females and males reported a history of heat exhaustion, suggesting that physical demand and heat stress may not completely account for the difference between sexes. We have no information on occupational exposure to agrochemicals, which could differ between sexes due to different tasks, crops, or individual practices (such as wearing personal protective equipment). Hyperuricemia was associated with CKDu, but longitudinal studies are needed to determine if this has a causative role in CKDu.

History of UTI remained a significant risk factor for traditional CKD in the multivariate analysis even when adjusting for multiple other factors. This may be due to the higher prevalence of diabetes in the traditional group, a major UTI risk factor [37]. Dysuria, which is frequently misdiagnosed as a UTI, has previously been associated with CKDu [13, 14]. However, in our study, the CKDu group had the lowest prevalence of current dysuria and had almost identical prevalence of UTI history as the no CKD group. In this population overall, both UTI history and dysuria were surprisingly common and warrant further investigation.

NSAID use did not differ significantly between groups, and diuretic use was highest in the traditional CKD group, which could be due to comorbid hypertension in this population. Overall, this fails to support the hypothesis that NSAID and diuretic use are important contributors to CKDu. Finally, family history was associated with 2 times the odds of traditional CKD but was not associated with CKDu, which does not necessarily support the hypothesis of a genetic contribution to CKDu risk. However, this is very difficult to examine directly since families also share many of the same exposures (drinking water source, socioeconomic status, etc.). Results were qualitatively similar in sensitivity analyses including participants with indeterminant CKD, who were excluded from the primary analyses.

Our study is one of few population-based analysis of CKD in areas endemic for CKDu that performed dual assessment of urine and serum. However, we acknowledge several limitations, including the single timepoint collection of kidney disease markers, when a clinical diagnosis of CKD requires abnormalities for at least 3 months. A large portion of the study population did not undergo urine testing and could have been misclassified. In addition, there is no eGFR equation that has been validated in the Nicaraguan population. We also relied on self-reported data, including history of traditional risk factors. There were no direct inquiries related to exposure to agrochemicals. It was assumed that all those with traditional risk factors who had CKD developed CKD due to these risk factors, when they could have CKDu or CKD from a different cause. The cross-sectional design prevents conclusions about the temporality between exposures and outcomes. Finally, the sample sizes for the individual CKD groups were small, although we were still able to detect important differences between groups.

## Conclusions

CKD has an alarmingly high prevalence of 8.6% in the municipality of León, Nicaragua in those less than 60 years old. Among participants with CKD, 35% lack traditional risk factors for disease and may have CKDu. Factors associated with suspected CKDu included male sex, well water source, and lower BMI. CKD from traditional causes was more strongly associated with family history of CKD, history of UTIs, and lower socioeconomic status. This study demonstrates a high burden of CKD from both traditional risk factors and CKDu in this region and emphasizes the importance of CKD screening. Future research should focus on identifying the cause of CKDu in order to implement strategies to prevent disease.

### Supplementary Information

**Additional file 1:** **Supplemental Figure 1.** Uric Acid by CKD Stage – Males Only. **Supplemental Figure 2.** Uric Acid by CKD Stage – Females Only. **Table S1.** Description and comparison of Indeterminant CKD group in demographics, health behaviors, medical history, and occupation. **Table S2.** Description and Comparison of Indeterminant CKD group in health assessment. **Table S3.** Sensitivity analysis: Indeterminant CKD group included as no CKD in demographics, health behaviors, medical history, and occupation. **Table S4.** Sensitivity analysis: Indeterminant CKD group included as no CKD in health assessment. **Table S5.** Odds ratios and 95% confidence intervals for the multinomial logistic regression model for CKD with traditional risk factors, suspected CKDu and Indeterminant CKD. **Table S6.** Odds ratios and 95% confidence intervals for the multinomial logistic regression model for CKD with traditional risk factors and suspected CKDu with the indeterminant CKD group counted as no CKD.

## Data Availability

The datasets used and/or analyzed during the current study available from the corresponding author on reasonable request.

## Abbreviations

CKD: Chronic kidney disease
CKDu: Chronic kidney disease of unknown etiology
NSAID: Non-steroidal anti-inflammatory drug
LHDSS: León Health and Demographic Surveillance System
CISTA: Research Center of Health, Work, and Environment (In Spanish, Centro de Investigación en Salud, Trabajo, y Ambiente)
CIDS: Center for Demography and Health Research (In Spanish, Centro de Investigación en Demografía y Salud)
UNAN: Universidad Nacional Autónoma de Nicaragua
BMI: Body mass index
RPM: Rotations per minute
MINSA: Nicaraguan Ministry of Health
BUN: Blood urea nitrogen
eGFR: Estimated glomerular filtration rate
KDIGO: Kidney Disease Improving Global Outcomes
PAHO: Pan American Health Organization
UTI: Urinary tract infection
OR: Odds ratio
CI: Confidence Interval

## References

[1] Foreman KJ, Marquez N, Dolgert A (2018). Forecasting life expectancy, years of life lost, and all-cause and cause-specific mortality for 250 causes of death: reference and alternative scenarios for 2016–40 for 195 countries and territories. Lancet.

[2] Jayasumana C, Orantes C, Herrera R (2017). Chronic interstitial nephritis in agricultural communities: a worldwide epidemic with social, occupational and environmental determinants. Nephrol Dial Transplant.

[3] Wijkström J, Leiva R, Elinder CG (2013). Clinical and pathological characterization of mesoamerican nephropathy: a new kidney disease in Central America. Am J Kidney Dis.

[4] González-Quiroz M, Pearce N, Caplin B, Nitsch D (2018). What do epidemiological studies tell us about chronic kidney disease of undetermined cause in Meso-America? a systematic review and meta-analysis. Clin Kidney J.

[5] Johnson RJ, Wesseling C, Newman LS (2019). Chronic kidney disease of unknown cause in agricultural communities. N Engl J Med.

[6] Milagres T, García-Arroyo FE, Lanaspa MA (2018). Rehydration with fructose worsens dehydration-induced renal damage. BMC Nephrol.

[7] Glaser J, Lemery J, Rajagopalan B (2016). Climate change and the emergent epidemic of CKD from heat stress in rural communities: the case for heat stress nephropathy. CJASN.

[8] Roncal-Jimenez C, García-Trabanino R, Barregard L (2016). Heat stress nephropathy from exercise-induced uric acid crystalluria: a perspective on mesoamerican nephropathy. Am J Kidney Dis.

[9] Holliday MW, Li Q, Bustamante EG (2022). Potential mechanisms involved in chronic kidney disease of unclear etiology. CJASN.

[10] Broe MED, Vervaet BA (2020). Is an Environmental Nephrotoxin the Primary Cause of CKDu (Mesoamerican Nephropathy)? PRO. Kidney360.

[11] Rango T, Jeuland M, Manthrithilake H, McCornick P (2015). Nephrotoxic contaminants in drinking water and urine, and chronic kidney disease in rural Sri Lanka. Sci Total Environ.

[12] Jayasumana C, Gajanayake R, Siribaddana S (2014). Importance of Arsenic and pesticides in epidemic chronic kidney disease in Sri Lanka. BMC Nephrol.

[13] Ramirez-Rubio O, Brooks DR, Amador JJ, Kaufman JS, Weiner DE, Scammell MK (2013). Chronic kidney disease in Nicaragua: a qualitative analysis of semi-structured interviews with physicians and pharmacists. BMC Public Health.

[14] Petropoulos ZE, Laws RL, Amador JJ (2020). Kidney function, self-reported symptoms, and urine findings in Nicaraguan sugarcane workers. Kidney360.

[15] Yang CW (2018). Leptospirosis renal disease: emerging culprit of chronic kidney disease unknown etiology. Nephron.

[16] Sunil-Chandra NP, Jayaweera JAAS, Kumbukgolla W, Jayasundara MVML (2020). Association of hantavirus infections and leptospirosis with the occurrence of chronic kidney disease of uncertain etiology in the North Central Province of Sri Lanka: a prospective study with patients and healthy persons. Front Cell Infect Microbiol.

[17] Siriwardhana EARIE, Perera PAJ, Sivakanesan R, Abeysekara T, Nugegoda DB, Jayaweera JAAS (2015). Dehydration and malaria augment the risk of developing chronic kidney disease in Sri Lanka. Indian J Nephrol.

[18] Isaranuwatchai S, Chanakul A, Ittiwut C (2021). Whole-exome sequencing solved over 2-decade kidney disease enigma. Nephron.

[19] Ordunez P, Nieto FJ, Martinez R (2018). Chronic kidney disease mortality trends in selected Central America countries, 1997–2013: clues to an epidemic of chronic interstitial nephritis of agricultural communities. J Epidemiol Community Health.

[20] Nicaragua: Administrative Division (Departments and Municipalities) - Population Statistics, Charts and Map. https://www.citypopulation.de/en/nicaragua/admin/. Accessed 21 Dec 2022.

[21] Peña R, Pérez W, Meléndez M, Källestål C, Persson LÅ (2008). The Nicaraguan Health and Demographic Surveillance Site, HDSS-León: A platform for public health research. Scand J Public Health.

[22] Ravallion M, Chen S, Sangraula P. Dollar a Day Revisited. Published online May 2008. 10.1596/1813-9450-4620.

[23] United Nations, ed. International Standard Industrial Classification of All Economic Activities (ISIC). Rev. 4. United Nations Statistical Papers; 2008. https://unstats.un.org/unsd/publication/seriesm/seriesm_4rev4e.pdf.

[24] Inker LA, Eneanya ND, Coresh J (2021). New creatinine- and cystatin C-based equations to estimate GFR without race. N Engl J Med.

[25] CKD Evaluation and Management – KDIGO. https://kdigo.org/guidelines/ckd-evaluation-and-management/. Accessed 21 Dec 2022.

[26] “Epidemic of Chronic Kidney Disease in Agricultural Communities in Central America. Case Definitions, Methodological Basis and Approaches for Public Health Surveillance.” Washington, D.C: Pan American Health Organization; 2017. https://iris.paho.org/handle/10665.2/34132.

[27] Sanchez Polo V, Garcia-Trabanino R, Rodriguez G, Madero M (2020). Mesoamerican Nephropathy (MeN): What we know so far. Int J Nephrol Renovasc Dis.

[28] R Core Team. R: A language and environment for statistical computing. Published online 2022. https://www.R-project.org/.

[29] Mayo Clinic Laboratories: Test Definition: URIC. Uric acid, serum. https://www.mayocliniclabs.com/api/sitecore/TestCatalog/DownloadTestCatalog?testId=8440. Accessed 1 July 2023.

[30] Gonzalez-Quiroz M, Smpokou ET, Silverwood RJ (2018). Decline in kidney function among apparently healthy young adults at risk of Mesoamerican nephropathy. JASN.

[31] Lebov JF, Valladares E, Peña R (2015). A population-based study of prevalence and risk factors of chronic kidney disease in León. Nicaragua Can J Kidney Health Dis.

[32] United States Renal Data System. 2016 USRDS Annual Data Report: Epidemiology of kidney disease in the United States. National Institutes of Health, National Institute of Diabetes and Digestive and Kidney Diseases, Bethesda, MD. 2016.

[33] Wegman DH, Apelqvist J, Bottai M (2018). Intervention to diminish dehydration and kidney damage among sugarcane workers. Scand J Work Environ Health.

[34] The Chronic Kidney Disease Epidemic in El Salvador: A Cross-Sectional Study. MEDICC Rev. 2019;21(2–3). 10.37757/MR2019.V21.N2-3.7. 10.37757/MR2019.V21.N2-3.7 31373582

[35] Vervaet BA, Nast CC, Jayasumana C (2020). Chronic interstitial nephritis in agricultural communities is a toxin-induced proximal tubular nephropathy. Kidney Int.

[36] Orantes Navarro CM, Almaguer López M, Alonso Galbán P, et al. The chronic kidney disease epidemic in El Salvador: the influence of agrochemicals. Rev Cuba Med Trop. 2020;72(2):e531. 10.37757/MR2019.V21.N2-3.7 31373582

[37] Joshi N, Caputo GM, Weitekamp MR, Karchmer AW (1999). Infections in Patients with Diabetes Mellitus. N Engl J Med.

