# Supplementary material for: Kidney disease characteristics, prevalence, and risk factors in León, Nicaragua: a population-based study
Source: BMC Nephrol. 2023 Nov 12;24:335. doi: 10.1186/s12882-023-03381-1 (PMC10641961; doi:10.1186/s12882-023-03381-1)
Supplement: Supplementary file 1 — Additional file 1: Supplemental Figure 1. Uric Acid by CKD Stage – Males Only. Supplemental Figure 2. Uric Acid by CKD Stage – Females Only. Table S1. Description and comparison of Indeterminant CKD group in demographics, health behaviors, medical history, and occupation. Table S2. Description and Comparison of Indeterminant CKD group in health assessment. Table S3. Sensitivity analysis: Indeterminant CKD group included as no CKD in demographics, health behaviors, medical history, and occupation. Table S4. Sensitivity analysis: Indeterminant CKD group included as no CKD in health assessment. Table S5. Odds ratios and 95% confidence intervals for the multinomial logistic regression model for CKD with traditional risk factors, suspected CKDu and Indeterminant CKD. Table S6. Odds ratios and 95% confidence intervals for the multinomial logistic regression model for CKD with traditional risk factors and suspected CKDu with the indeterminant CKD group counted as no CKD. [file 12882_2023_3381_MOESM1_ESM.docx]

**Supplemental Materials**

Supplemental Figures

Supplemental Tables

**Supplemental Figures**

**Supplemental Figure 1: Uric Acid by CKD Stage – Males Only.**


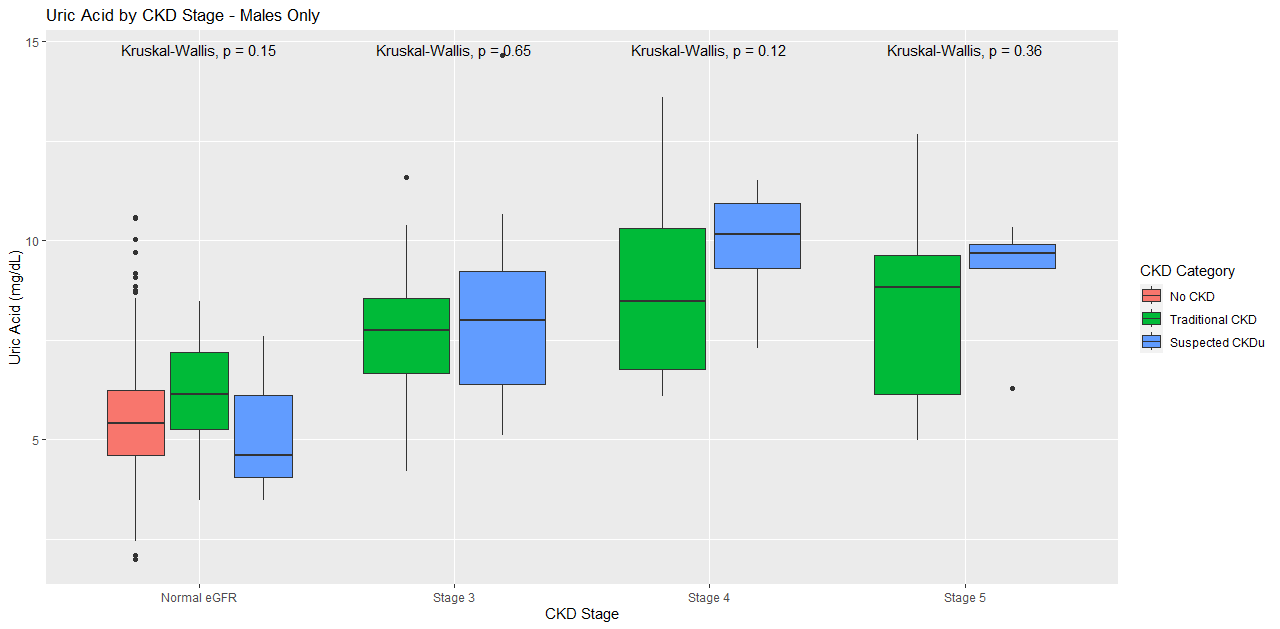


**Footnote:** None of the uric acid level differences between CKD categories were statistically significant. P-values for Kruskal Wallis test comparing uric acid levels between CKD groups at each stage of CKD were <0.05.


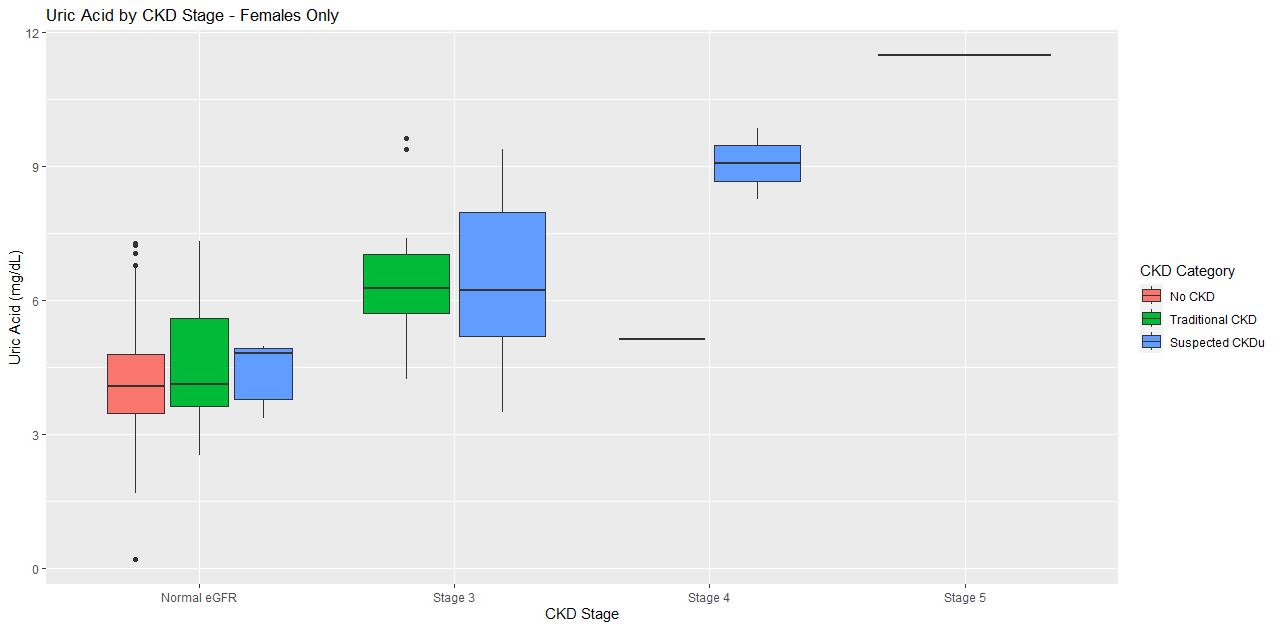


**Supplemental Figure 2: Uric Acid by CKD Stage – Females Only.**

**Footnote:** Kruskal Wallis test only able to be performed on normal eGFR and Stage 3 groups due to sample size. For normal eGFR groups, p=0.501. For stage 3, p=0.935. All were not significant (p>0.05).

**Supplemental Tables:**

**Table S1:** Description and comparison of Indeterminant CKD group in demographics, health behaviors, medical history, and occupation

| Characteristics | No CKD (n=1289) | CKD with traditional risk factors (n=100) | Suspected CKDu group  (n= 55) | Indeterminant group (n=351) | P value |
| --- | --- | --- | --- | --- | --- |
| Demographics |  |  |  |  |  |
| Sex |  |  |  |  | <0.001^a^ * |
| Male | 513 (39.8%) | 48 (48.0%) | 37 (67.3%) | 112 (31.9%) |  |
| Female | 776 (60.2%) | 52 (52.0%) | 18 (32.7%) | 239 (68.1%) |  |
| Age, median (IQR) | 33 (23-43) | 48 (37-54) | 44 (34-55) | 42 (30-51) | <0.001^b^ * |
| Socioeconomic level |  |  |  |  | 0.053^a^ ^&^ |
| Extremely poor | 421 (33.0%) | 43 (43.0%) | 24 (43.6%) | 107 (30.7%) |  |
| Poor | 328 (25.7%) | 29 (29.0%) | 13 (23.6%) | 92 (26.4%) |  |
| Not poor | 527 (41.3%) | 28 (28.0%) | 16 (29.1%) | 150 (43.0%) |  |
| Illiterate | 75 (5.8%) | 13 (13.0%) | 11 (20.0%) | 31 (8.8%) | <0.001^a^ * |
| Highest grade level completed, median (IQR)^c^ | 9 (6-12) | 6 (3-11) | 6 (4-9) | 9 (6-11) | <0.001^a^ * |
| Rural Zone | 447 (34.7%) | 37 (37.0%) | 24 (43.6%) | 111 (31.6%) | 0.301^a^ |
| Well water source^d^ | 242 (18.8%) | 29 (29.0%) | 21 (38.2%) | 78 (22.3%) | <0.001^a^ * |
| Health behaviors |  |  |  |  |  |
| Daily water intake (L), median (IQR) | 2 (1-3) | 2 (1-4) | 3 (2-4) | 2 (1-3) | 0.046^b^ * |
| Daily soda intake (L), median (IQR) | 0.25 (0-0.5) | 0 (0 – 0.5) | 0 (0-0.5) | 0 (0-0.5) | 0.006^b*&^ |
| Alcohol consumption in last day | 80 (6.2%) | 2 (2.0%) | 7 (12.7%) | 18 (5.1%) | 0.124^b^ |
| History of smoking | 285 (22.1%) | 27 (27.0%) | 19 (34.5%) | 73 (20.8%) | 0.097^a^ |
| Median years smoking | 8 (3-19) | 22 (13-35) | 24 (16-35) | 16 (4-28) | <0.001^b^ * |
| History of drug consumption | 121 (9.4%) | 12 (12.0%) | 6 (10.9%) | 27 (7.7%) | 0.495^a^ |
| Exercise ≥ 2 times weekly | 256 (19.9%) | 14 (14.0%) | 7 (12.7%) | 56 (16.0%) | 0.149^a^ |
| Eat fruits, vegetables, or salads ≥ 2 times weekly | 769 (59.9%) | 65 (60.0%) | 23 (41.8%) | 202 (58.0%) | 0.063^a &^ |
| Medical history |  |  |  |  |  |
| NSAID use | 346 (26.8%) | 37 (37.0%) | 16 (29.1%) | 107 (30.5%) | 0.107^a^ |
| Diuretic use | 49 (3.8%) | 13 (13.0%) | 2 (3.6%) | 19 (5.4%) | 0.002^a^ * |
| Hypertension | 142 (11.0%) | 53 (53.0%) | 0 | 77 (21.9%) | <0.001^a^ * |
| Diabetes | 52 (4.0%) | 22 (22.0%) | 0 | 31 (8.8%) | <0.001^a^ * |
| Kidney stones | 67 (5.2%) | 18 (18.0%) | 0 | 28 (8.0%) | <0.001^a^ * |
| Arthritis | 106 (8.2%) | 27 (27.0%) | 8 (14.5%) | 58 (16.5%) | <0.001^a^ * |
| Malaria | 128 (9.9%) | 15 (15.0%) | 7 (12.7%) | 39 (11.1%) | 0.336^a^ |
| Urinary tract infection | 631 (49.0%) | 65 (65.0%) | 28 (50.1%) | 185 (52.9%) | 0.016^a^ ^*^ |
| Family history of CKD | 312 (24.2%) | 45 (41.3%) | 13 (28.3%) | 95 (27.1%) | 0.002^a^ * |
| Occupation |  |  |  |  |  |
| Current agricultural occupation^e^ | 86 (6.7%) | 11 (11.0%) | 16 (29.1%) | 23 (6.6%) | <0.001^a^ * |
| History of working in agriculture | 228 (17.7%) | 27 (27.0%) | 25 (45.5%) | 69 (19.7%) | <0.001^a^ * |
| Males | 152 (66.7%) | 24 (88.9%) | 22 (88.0%) | 39 (56.5%) | <0.001^a^ * |
| Females | 76 (33.3%) | 3 (10.7%) | 3 (12.5%) | 30 (43.5%) | 0.329^a^ |
| Years worked, median(IQR) | 10 (5-20) | 13 (9-30) | 20 (8-32) | 14 (5-24) | 0.040^b^ * |
| History of fainting at work | 136 (10.6%) | 17 (17.0%) | 6 (10.9%) | 39 (11.1%) | 0.271^a^ |
| History of heat exhaustion | 445 (34.5%) | 42 (42.0%) | 15 (27.2%) | 114 (32.5%) | 0.231^a^ |

**Footnotes:** All percentages calculated from non-missing data. Less than 5% of each group is missing data unless otherwise indicated. a. p-value calculated with Fisher’s Exact Test. b. p-value calculated with Kruskal Wallis test. c. 124 participants missing grade level (69 in no CKD, 14 in traditional CKD, 9 in suspected CKDu, 32 in indeterminant group). D. The remainder of participants obtained water from a public water system with interior plumping with the exception of 4 participants in the No CKD group who had a different water source. E. Of those with a current occupation in agriculture, all were male except 4 females in the no CKD group and 6 females in the indeterminant group.

* significant finding at p-value <0.05. & statistical significance different from analysis included in manuscript

Abbreviations: CKD: Chronic kidney disease. CKDu: Chronic kidney disease of unknown etiology. IQR: Interquartile range. L: Liters. NSAID: Non-steroidal anti-inflammatory drug.

| Health assessment | No CKD (n=1289) | CKD with traditional risk factors (n=100) | Suspected CKDu group (n= 55) | Indeterminant group (n=351) | P value |
| --- | --- | --- | --- | --- | --- |
| BMI categories |  |  |  |  | 0.524^a^ |
| Underweight (<18.5) | 46 (3.6%) | 3 (3.0%) | 1 (1.8%) | 10 (2.9%) |  |
| Normal (18.5 – 24.5) | 427 (33.4%) | 28 (28.0%) | 28 (50.1%) | 88 (25.1%) |  |
| Overweight (25-30) | 442 (34.6%) | 38 (38.0%) | 14 (25.4%) | 118 (33.7%) |  |
| Obese (30-34.5) or extremely obese (>35) | 363 (28.4%) | 33 (33.0%) | 10 (18.1%) | 134 (38.3%) |  |
| BMI, median (IQR) | 27 (23-31) | 28 (24-31) | 24 (22-28) | 28 (24-32) | <0.001^b^ * |
| Serum uric acid (umol/L), median (IQR) |  |  |  |  |  |
| Males (reference range 220-476)^c^ | 321 (274 – 371) | 435 (360 - 508) | 523 (430 – 589) | 362 (312 – 424) | <0.001^b^ * |
| Females (reference range 161-363)^c^ | 243 (206 – 249) | 335 (245 – 400) | 359 (288 – 500) | 275 (234-324) | <0.001^b^ * |
| Serum BUN (mmol/L), median (IQR) | 3.0 (2.4 – 3.7) | 5.5 (3.9 – 9.0) | 7.1 (5.5 – 9.4) | 3.5 (2.7 – 4.3) | <0.001^b^ * |
| Serum Creatinine (umol/L), median (IQR) | 59 (51 - 69) | 127 (69 – 184) | 172 (127 -236) | 71 (55 – 87) | <0.001^b^ * |
| eGFR (ml/min/1.73 m^2^), median (IQR) | 120 (110-129) | 51 (36-105) | 40 (25-54) | 105 (81-121) | <0.001^b^ * |
| CKD Stages |  |  |  |  | <0.001^a^ * |
| Proteinuria with eGFR > 60 ml/min/1.73 m^2^ | 0 | 39 (39.0%) | 8 (14.5%) | 0 |  |
| Stage 3 | 0 | 42 (42.0%) | 29 (52.7%) | 0 |  |
| Stage 4 | 0 | 12 (12.0%) | 13 (23.6%) | 0 |  |
| Stage 5 | 0 | 7 (7.0%) | 5 (9.1%) | 0 |  |
| Urine dipstick results |  |  |  |  |  |
| Positive leukocyte esterase^d^ | 144 (14.3%) | 25 (25.5%) | 7 (17.5%) | 81 (25.6%) | <0.001^a^ * |
| Positive nitrites^e^ | 44 (4.3%) | 10 (9.9%) | 1 (2.5%) | 43 (13.5%) | <0.001^a^ * |
| Proteinuria^f^ |  |  |  |  | <0.001^a^ * |
| None | 993 (100%) | 39 (41.1%) | 31 (77.5%) | 206 (64.9%) |  |
| Trace | 0 | 8 (8.4%) | 9 (22.5%) | 111 (35.1%) |  |
| 30 mg/dl | 0 | 5 (5.3%) | 7 (14.3%) | 0 |  |
| 100 mg/dl | 0 | 2 (2.1%) | 2 (4.1%) | 0 |  |
| 300 mg/dl | 0 | 36 (37.9%) | 0 | 0 |  |
| 1000 mg/dl |  | 5 (5.3%) | 0 | 0 |  |
| Hematuria^g^ | 0 | 12 (12.5%) | 0 | 111 (32.9%) | <0.001^a^ * |
| Current symptoms |  |  |  |  |  |
| High thirst | 676 (52.4%) | 57 (57.0%) | 28 (50.1%) | 172 (49.1%) | 0.518^a^ |
| Dysuria | 351 (27.2%) | 28 (28.0%) | 9 (16.4%) | 95 (27.2%) | 0.349^a^ |
| Dark urine | 356 (27.6%) | 36 (36.0%) | 8 (14.5%) | 97 (27.8%) | 0.038^a^ * |
| Cramps | 241 (18.7%) | 37 (37.0%) | 16 (29.1%) | 97 (27.8%) | <0.001^a^ * |

**Table S2:** Description and Comparison of Indeterminant CKD group in health assessment **Footnotes:** All percentages calculated from non-missing data. Less than 5% of each group is missing data unless otherwise indicated. a. p-value calculated with Fisher’s Exact Test. b. p-value calculated with Kruskal Wallis test. c. Reference range from Mayo Clinic Reference Laboratories and units converted.^28^ d. 333 participants missing leukocyte esterase e. 313 participants missing nitrites f. 342 participants missing proteinuria data. g. 309 participants missing hematuria data. * significant finding at p-value <0.05.

Abbreviations: CKD: Chronic kidney disease. CKDu: Chronic kidney disease of unknown etiology. BMI: body mass index. IQR: Interquartile range. BUN: blood urea nitrogen.

**Table S3:** Sensitivity analysis: Indeterminant CKD group included as no CKD in demographics, health behaviors, medical history, and occupation

| Characteristics | No CKD (with indeterminants included) (n=1640) | CKD with traditional risk factors (n=100) | Suspected CKDu group  (n= 55) | P value |
| --- | --- | --- | --- | --- |
| Demographics |  |  |  |  |
| Sex |  |  |  | <0.001^a^ * |
| Male | 625 (38.1%) | 48 (48.0%) | 37 (67.3%) |  |
| Female | 1015 (61.9%) | 52 (52.0%) | 18 (32.7%) |  |
| Age, median (IQR) | 34 (24-45) | 48 (37-54) | 44 (34-55) | <0.001^b^ * |
| Socioeconomic level^1^ |  |  |  | 0.019^a*^ |
| Extremely poor | 528 (32.5%) | 43 (43.0%) | 24 (43.6%) |  |
| Poor | 420 (25.8%) | 29 (29.0%) | 13 (23.6%) |  |
| Not poor | 677 (41.7%) | 28 (28.0%) | 16 (29.1%) |  |
| Illiterate | 106 (6.5%) | 13 (13.0%) | 11 (20.0%) | <0.001^a^ * |
| Highest grade level completed, median (IQR)^c^ | 9 (6-12) | 6 (3-11) | 6 (4-9) | <0.001^a^ * |
| Rural Zone | 558 (34.0%) | 37 (37.0%) | 24 (43.6%) | 0.286^a^ |
| River or well water source^d^ | 320 (19.6%) | 29 (29.0%) | 21 (38.2%) | <0.001^a^ * |
| Health behaviors |  |  |  |  |
| Daily water intake (L), median (IQR) | 2 (1-3) | 2 (1-4) | 3 (2-4) | 0.003^b^ * |
| Daily soda intake (L), median (IQR) | 0.25 (0-0.5) | 0 (0 – 0.5) | 0 (0-0.5) | 0.805^b^ |
| Alcohol consumption in last day | 98 (6.0%) | 2 (2.0%) | 7 (12.7%) | 0.081^b^ |
| History of smoking | 358 (21.8%) | 27 (27.0%) | 19 (34.5%) | 0.047^a*&^ |
| Median years smoking | 9 (3-23) | 22 (13-35) | 24 (16-35) | <0.001^b^ * |
| History of drug consumption | 148 (9.0%) | 12 (12.0%) | 6  (10.9%) | 0.457^a^ |
| Exercise ≥ 2 times weekly | 312 (19.1%) | 14 (14.0%) | 7 (12.7%) | 0.277^a^ |
| Eat fruits, vegetables, or salads ≥ 2 times weekly | 971 (59.5%) | 60 (60.0%) | 23 (41.8%) | 0.032^a^ * |
| Medical history |  |  |  |  |
| NSAID use | 453 (27.7%) | 37 (37.0%) | 16 (29.1%) | 0.115^a^ |
| Diuretic use | 68 (4.1%) | 13 (13.0%) | 2 (3.6%) | 0.001^a^ * |
| Hypertension | 219 (13.4%) | 53 (53.0%) | 0 | <0.001^a^ * |
| Diabetes | 83 (5.1%) | 22 (22.0%) | 0 | <0.001^a^ * |
| Kidney stones | 95 (5.8%) | 18 (18.0%) | 0 | <0.001^a^ * |
| Arthritis | 164 (10%) | 27 (27.0%) | 8 (14.5%) | <0.001^a^ * |
| Malaria | 167 (10.2%) | 15 (15.0%) | 7 (12.7%) | 0.224^a^ |
| Urinary tract infection | 816 (49.8%) | 65 (65.0%) | 28 (50.1%) | 0.013^a *^ |
| Family history of CKD | 407 (24.8%) | 42 (42.0%) | 16 (29.1%) | <0.001^a^ * |
| Occupation |  |  |  |  |
| Current agricultural occupation^e^ | 109 (6.6%) | 11 (11.0%) | 16 (29.1%) | <0.001^a^ * |
| History of working in agriculture | 297 (18.1%) | 27 (27.0%) | 25 (45.5%) | <0.001^a^ * |
| Males | 191 (64.3%) | 24 (88.9%) | 22 (88.0%) | <0.001^a^ * |
| Females | 106 (35.7%) | 3 (11.1%) | 3 (12.5%) | 0.412^a^ |
| Years worked, median(IQR) | 10 (5-20) | 13 (9-30) | 20 (8-32) | 0.206^b^ ^&^ |
| History of fainting at work | 175 (10.7%) | 17 (17.0%) | 6 (10.9%) | 0.146^a^ |
| History of heat exhaustion | 559 (34.1%) | 42 (42.0%) | 15 (27.2%) | 0.154^a^ |

**Footnotes:** All percentages calculated from non-missing data. Less than 5% of each group is missing data unless otherwise indicated. a. p-value calculated with Fisher’s Exact Test. b. p-value calculated with Kruskal Wallis test. c. 124 participants missing grade level. D. The remainder of participants obtained water from a public water system with interior plumping with the exception of 4 participants in the No CKD group who had a different water source. E. Of those with a current occupation in agriculture, all were male except 10 females in the no CKD group.

* significant finding at p-value <0.05. & statistical significance different from analysis included in manuscript

Abbreviations: CKD: Chronic kidney disease. CKDu: Chronic kidney disease of unknown etiology. IQR: Interquartile range. L: Liters. NSAID: Non-steroidal anti-inflammatory drug.

| Health assessment | No CKD (with indeterminants included) (n=1640) | CKD with traditional risk factors (n=100) | Suspected CKDu group (n= 55) | P value |
| --- | --- | --- | --- | --- |
| BMI categories |  |  |  | 0.029^a *^ |
| Underweight (<18.5) | 56 (3.4%) | 3 (3.0%) | 1 (1.8%) |  |
| Normal (18.5 – 24.5) | 515 (31.6%) | 28 (28.0%) | 28 (50.1%) |  |
| Overweight (25-30) | 560 (34.4%) | 38 (38.0%) | 14 (25.4%) |  |
| Obese (30-34.5) or extremely obese (>35) | 497 (30.5%) | 33 (33.0%) | 10 (18.1%) |  |
| BMI, median (IQR) | 27 (23-31) | 28 (24-31) | 24 (22-28) | 0.004^b^ * |
| Serum uric acid (umol/L), median (IQR) |  |  |  |  |
| Males (reference range 220-476)^c^ | 327 (280 – 380) | 435 (360 - 508) | 523 (430 – 589) | <0.001^b^ * |
| Females (reference range 161-363)^c^ | 249 (211 – 296) | 335 (245 – 400) | 359 (288 – 500) | <0.001^b^ * |
| Serum BUN (mmol/L), median (IQR) | 3.1 (2.5 – 3.8) | 5.5 (3.9 – 9.0) | 7.1 (5.5 – 9.4) | <0.001^b^ * |
| Serum Creatinine (umol/L), median (IQR) | 69 (58 - 80) | 127 (69 – 184) | 172 (127 -236) | <0.001^b^ * |
| eGFR (ml/min/1.73 m^2^), median (IQR) | 118 (107-128) | 51 (36-105) | 40 (25-54) | <0.001^b^ * |
| CKD Stages |  |  |  | <0.001^a^ * |
| Proteinuria with eGFR > 60 ml/min/1.73 m^2^ | 0 | 39 (39.0%) | 8 (14.5%) |  |
| Stage 3 | 0 | 42 (42.0%) | 29 (52.7%) |  |
| Stage 4 | 0 | 12 (12.0%) | 13 (23.6%) |  |
| Stage 5 | 0 | 7 (7.0%) | 5 (9.1%) |  |
| Urine dipstick results |  |  |  |  |
| Positive leukocyte esterase^d^ | 225 (17.0%) | 20 (20.8%) | 12 (24.5%) | 0.218^a &^ |
| Positive nitrites^e^ | 87 (6.5%) | 7 (7.3%) | 4 (8.1%) | 0.726^a^ |
| Proteinuria^f^ |  |  |  | <0.001^a^ * |
| None | 1198 (91.5%) | 39 (41.1%) | 31 (63.2%) |  |
| Trace | 111 (8.5%) | 8 (8.4%) | 9 (18.4%) |  |
| 30 mg/dl | 0 | 5 (5.3%) | 7 (14.3%) |  |
| 100 mg/dl | 0 | 2 (2.1%) | 2 (4.1%) |  |
| 300 mg/dl | 0 | 36 (37.9%) | 0 |  |
| 1000 mg/dl | 0 | 5 (5.3%) | 0 |  |
| Current symptoms |  |  |  |  |
| High thirst | 848 (51.7%) | 57 (57.0%) | 28 (50.1%) | 0.590^a^ |
| Dysuria | 446 (27.2%) | 28 (28.0%) | 9 (16.4%) | 0.198^a^ |
| Dark urine | 453 (27.6%) | 36 (36.0%) | 8 (14.5%) | 0.015^a^ * |
| Cramps | 338 (20.6%) | 37 (37.0%) | 16 (29.1%) | <0.001^a^ * |

**Table S4:** Sensitivity analysis: Indeterminant CKD group included as no CKD in health assessment

**Footnotes:** All percentages calculated from non-missing data. Less than 5% of each group is missing data unless otherwise indicated. a. p-value calculated with Fisher’s Exact Test. b. p-value calculated with Kruskal Wallis test. c. Reference range from Mayo Clinic Reference Laboratories and units converted.^28^ d. 333 participants missing leukocyte esterase data e. 313 participants missing nitrites data f. 342 participants missing proteinuria.

* significant finding at p-value <0.05. & statistical significance different from analysis included in manuscript.

Abbreviations: CKD: Chronic kidney disease. CKDu: Chronic kidney disease of unknown etiology. BMI: body mass index. IQR: Interquartile range. BUN: blood urea nitrogen.

**Table S5:** Odds ratios and 95% confidence intervals for the multinomial logistic regression model for CKD with traditional risk factors, suspected CKDu and Indeterminant CKD.

| **Independent Variable** | **Odds Ratio (95% Confidence Interval) Compared to those without CKD** | | | |
| --- | --- | --- | --- | --- |
|  | | **CKD with traditional risk factors** | **Suspected CKDu** | **Indeterminant CKD** |
|  | |  |  |  |
| Male sex | | 2.63 (1.63-4.24) | 2.89 (1.50-5.58) | 0.81 (0.61-1.08) |
| Age (10 year increase) | | 2.32 (1.90-2.84) | 2.10 (1.63-2.71) | 1.51 (1.36-1.68) |
| Lower socioeconomic level | | 1.33 (1.03-1.73) | 1.21 (0.86-1.71) | 0.91 (0.78-1.05) |
| Family History of CKD | | 2.01 (1.29-3.11) | 1.23 (0.67-2.32) | 1.06 (0.80-1.40) |
| History of urinary tract infections | | 1.79 (1.13-2.82) | 1.09 (0.61-1.96) | 1.03 (0.80-1.32) |
| Well water source^1^ | | 2.10 (1.23-3.59) | 2.20 (1.12-4.35) | 1.49 (1.07-2.06) |
| BMI (per 5 unit increase) | | 0.94 (0.78-1.14) | 0.59 (0.44-0.82) | 1.06 (0.97-1.15) |
| History of working in agriculture | | 0.52 (0.29-0.92) | 1.18 (0.61-1.96) | 0.84 (0.58-1.20) |

**Footnotes:** 1. Well or river source versus public water system with interior plumbing. Variance inflation factors all <1.4.

Abbreviations: CKD: Chronic kidney disease. CKDu: CKD from unknown etiology. BMI: Body mass index.

**Table S6:** Odds ratios and 95% confidence intervals for the multinomial logistic regression model for CKD with traditional risk factors and suspected CKDu with the indeterminant CKD group counted as no CKD.

| **Independent Variable** | **Odds Ratio (95% Confidence Interval) Compared to those without CKD (+indeterminant group)** | |
| --- | --- | --- |
|  | **CKD with traditional risk factors** | **Suspected CKDu** |
|  |  |  |
| Male sex | 2.30 (1.45-3.56) | 3.27 (1.77-6.04) |
| Age (10 year increase) | 2.00 (1.65-2.43) | 1.96 (1.54-2.49) |
| Lower socioeconomic level | 1.33 (1.03-1.71) | 1.26 (0.89-1.76) |
| Family History of CKD | 1.91 (1.24-2.94) | 1.25 (0.67-2.32) |
| History of urinary tract infections | 1.74 (1.11-2.72) | 1.09 (0.61 – 1.96) |
| Well water source^1^ | 1.52 (0.93-2.48) | 2.20 (1.20-4.07) |
| BMI (per 5 unit increase) | 0.93 (0.78-1.12) | 0.59 (0.43-0.81) |

**Footnotes:** 1. Well or river source versus public water system with interior plumbing. Variance inflation factors all <1.2.

Abbreviations: CKD: Chronic kidney disease. CKDu: CKD from unknown etiology. BMI: Body mass index.
